# Epigenetic suppression of neprilysin regulates breast cancer invasion

**DOI:** 10.1038/oncsis.2016.16

**Published:** 2016-03-07

**Authors:** H M Stephen, R J Khoury, P R Majmudar, T Blaylock, K Hawkins, M S Salama, M D Scott, B Cosminsky, N K Utreja, J Britt, R E Conway

**Affiliations:** 1 Lipscomb University, Department of Biology, College of Liberal Arts and Science, 1 University Park Drive, Nashville, TN, USA

## Abstract

In women, invasive breast cancer is the second most common cancer and the second cause of cancer-related death. Therefore, identifying novel regulators of breast cancer invasion could lead to additional biomarkers and therapeutic targets. Neprilysin, a cell-surface enzyme that cleaves and inactivates a number of substrates including endothelin-1 (ET1), has been implicated in breast cancer, but whether neprilysin promotes or inhibits breast cancer cell progression and metastasis is unclear. Here, we asked whether neprilysin expression predicts and functionally regulates breast cancer cell invasion. RT–PCR and flow cytometry analysis of MDA-MB-231 and MCF-7 breast cancer cell lines revealed decreased neprilysin expression compared with normal epithelial cells. Expression was also suppressed in invasive ductal carcinoma (IDC) compared with normal tissue. In addition, *in vtro* invasion assays demonstrated that neprilysin overexpression decreased breast cancer cell invasion, whereas neprilysin suppression augmented invasion. Furthermore, inhibiting neprilysin in MCF-7 breast cancer cells increased ET1 levels significantly, whereas overexpressing neprilysin decreased extracellular-signal related kinase (ERK) activation, indicating that neprilysin negatively regulates ET1-induced activation of mitogen-activated protein kinase (MAPK) signaling. To determine whether neprilysin was epigenetically suppressed in breast cancer, we performed bisulfite conversion analysis of breast cancer cells and clinical tumor samples. We found that the neprilysin promoter was hypermethylated in breast cancer; chemical reversal of methylation in MDA-MB-231 cells reactivated neprilysin expression and inhibited cancer cell invasion. Analysis of cancer databases revealed that neprilysin methylation significantly associates with survival in stage I IDC and estrogen receptor-negative breast cancer subtypes. These results demonstrate that neprilysin negatively regulates the ET axis in breast cancer, and epigenetic suppression of neprilysin in invasive breast cancer cells enables invasion. Together, this implicates neprilysin as an important regulator of breast cancer invasion and clarifies its utility as a potential biomarker for invasive breast cancer.

## Introduction

Neprilysin/neutral endopeptidase 24.11 (NEP), also known as membrane metallo-endopeptidase, CD10 and common acute lymphoblastic leukemia antigen,^1^ is a 95–100 kDa cell-surface endopeptidase that cleaves and inactivates numerous peptide substrates at hydrophobic amino acids. NEP substrates include β-amyloid, angiotensin, bradykinin, substance P and endothelin-1 (ET1) (reviewed by LeBien and McCormack,^2^ Gafford *et al.*,^3^ Johnson *et al.*^4^ and Vijayaraghavan *et al.*^5^). ET1 activates the mitogen-activated protein kinase (MAPK) pathway through ET receptor signaling and subsequently modulates cell survival, proliferation, invasion and angiogenesis (reviewed by Rosano *et al.*^6^) and is implicated in numerous cancers.

Despite its apparent functional inhibition of ET signaling by ET1 inactivation, clinical and experimental data of NEP in various cancers are conflicting. Although several reports suggest a protective role for NEP in solid cancers, including breast cancer,^7, 8, 9, 10, 11, 12^ studies in a number of solid cancers including colorectal, head and neck squamous cell carcinoma, lung cancer and also breast cancer, implicate NEP as a possible marker for tumor progression and metastasis.^13, 14, 15, 16, 17, 18, 19, 20, 21, 22, 23, 24, 25^ Despite the abundant literature detailing the contribution of the ET axis to breast cancer and NEP's ability to regulate ET signaling,^26, 27, 28, 29, 30, 31, 32^ the specific function of NEP and its mechanism in breast cancer invasion remains unclear.

Our lab is interested in studying the mechanisms controlling breast cancer invasion, as a clearer understanding of the genes and proteins modulating this process could reveal potential therapeutic targets for treating invasive breast cancer. Here, we report decreased expression of NEP in breast cancer cell lines and primary tumor samples, accompanied by increased NEP promoter methylation. In addition, we demonstrate that NEP acts as an inhibitor of breast cancer cell invasion by negatively regulating ET1-mediated signaling. Finally, we show that NEP methylation may be a useful prognostic biomarker for stage I and estrogen receptor (ER)-negative invasive ductal carcinoma (IDC). Together, these results implicate NEP as a potential biomarker and important regulator of breast cancer cell invasion.

## Results

### NEP expression in breast cancer cell lines

**Figure 1:**
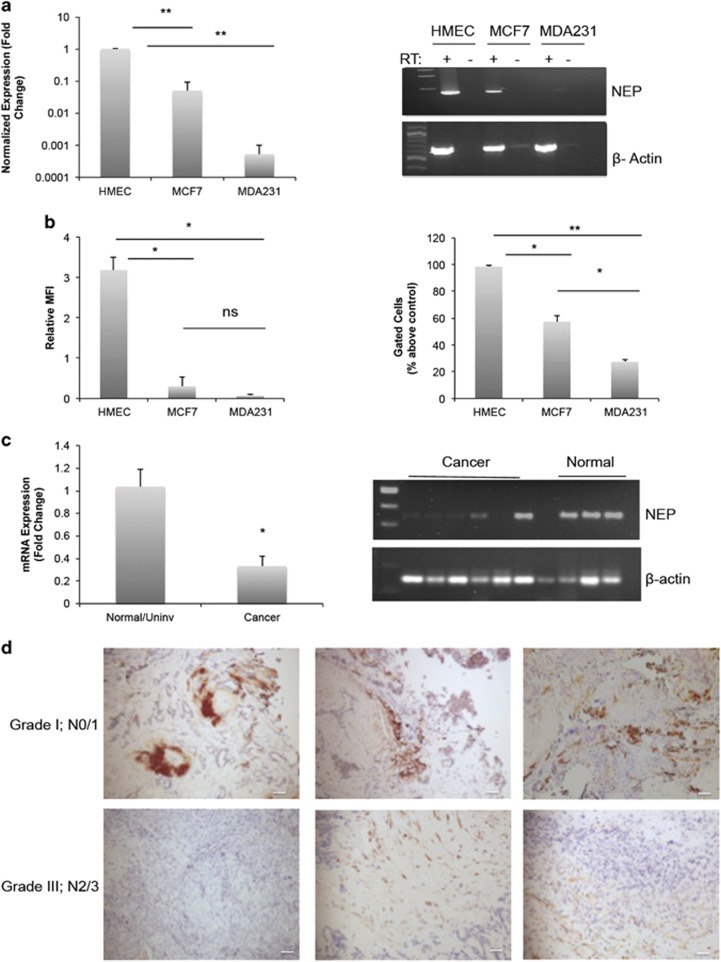
NEP expression in breast cancer cells. (**a**) Left: quantitative RT–PCR was performed with cDNA from HMEC, MCF-7 and MDA-MB-231 cells; average fold-change over HMEC values of biological triplicates are shown (*P*=0.0015 for HMEC vs MCF-7; *P*=0.0002 for HMEC vs MDA-MB-231; MCF-7 vs MDA-MB-231 was not significant). Right: representative gel of end point RT–PCR (RT+) and no-RT controls (RT−). The gel shown is cropped to the relevant molecular weight. (**b**) Graphs quantifying median fluorescent intensities (left) (HMEC vs MCF-7 *P*=0.018; HMEC vs MDA-MB-231 *P*=0.01) and percent gated cells (right) (HMEC vs MCF-7 *P*=0.0013; HMEC vs MDA-MB-231 *P*<0.001; MCF-7 vs MDA-MB-231 *P*=0.025) from three independent flow cytometry experiments. (**c**) Left: quantitative RT–PCR of RNA extracted from human IDC samples (*n*=7) demonstrates significantly reduced NEP mRNA compared with normal and uninvolved breast tissue (*n*=4); *P*=0.01. Representative Ct and melt curve graphs are shown in Supplementary Figure 2. Right: representative gel of end point RT–PCR of NEP amplified from IDC and normal cDNA. Gels are cropped to the appropriate molecular weight. No RT/PCR controls are shown in Supplementary Figure 2. (**d**) Representative NEP immunohistochemistry images from low grade, N0/N1 IDC (top) and high grade, metastatic (N2/3) IDC tumor samples.

On the basis of previously characterized functions of NEP in other cells and tissues, we initially hypothesized that NEP would be suppressed in invasive breast cancer cell lines. To specifically address this, we performed quantitative RT–PCR analysis of NEP expression in primary mammary cells (human mammary epithelial cells, HMECs) and breast cancer cell lines (MDA-MB-231 and MCF-7). We found that NEP mRNA is significantly decreased in both cancer cell lines compared with the normal cells (Figure 1a). Protein expression analysis by flow cytometry demonstrates high expression in normal breast cells, but its expression is notably lower in the breast cancer cell lines, with the invasive MDA-MB-231 cells expressing the lowest levels of NEP protein (Figure 1b). To verify our observations in clinically relevant tissue, we measured NEP expression by quantitative RT–PCR in human IDC samples and normal or uninvolved breast tissue. NEP mRNA was significantly lower in IDC samples compared with normal controls (Figure 1c, Supplementary Figure 2). Consistent with previously published reports,^33, 34^ immunohistochemical analysis of NEP expression in low-grade IDC revealed strong epithelial staining, whereas NEP expression in high grade, metastatic IDC samples was notably lower and largely confined to the stroma (Figure 1d).

### NEP negatively regulates invasion

**Figure 2:**
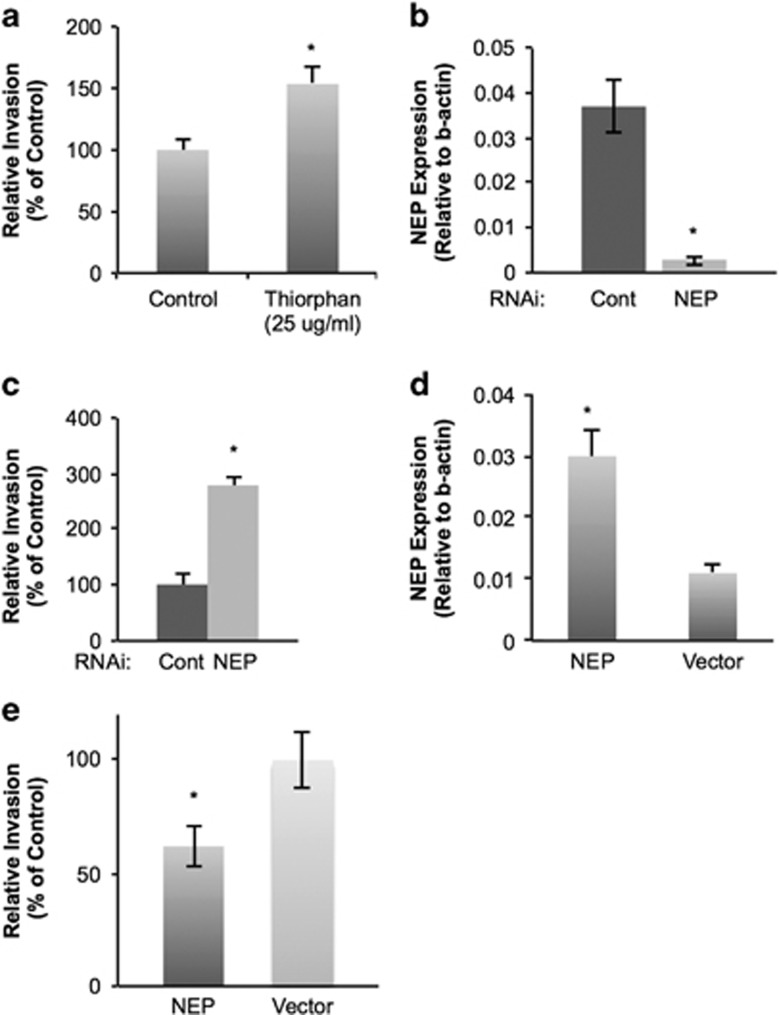
NEP negatively regulates breast cancer invasion. (**a**) Averages of three independent MCF-7 *in vitro* invasion assays in the presence or absence of 25 μg/ml thiorphan, a NEP-specific inhibitor, demonstrate increased invasion in the presence of thiorphan (*P*=0.025). Cell viability is not altered in the presence of thiorphan (Supplementary Figure 3). (**b**) Transfecting MCF-7 cells with NEP RNAi results in a significant decrease of NEP mRNA as measured by RT–PCR. Averages of semi-quantitative analysis of three independent experiments normalized to β-actin are shown (*P*=0.02). (**c**) *In vitro* invasion assays with MCF-7 cells transfected with control siRNA or NEP siRNA show significantly increased adhesion when NEP is knocked down (*P*=0.0004 from three independent experiments). (**d**) Transfecting an expression vector encoding NEP into MDA-MB-231 cells results in increased mRNA expression compared with cells transfected with vector only as demonstrated by RT–PCR (*P*=0.035). (**e**) MDA-MB-231 cells transfected with NEP plasmid exhibited significantly less invasion than cells transfected with the control plasmid (*P*=0.024 from three independent experiments).

After observing decreased NEP expression in breast cancer cells and IDC, we next hypothesized that NEP negatively regulates breast cancer cell invasion. To initially test this, we measured MCF-7 cell invasion in the presence and absence of the NEP inhibitor thiorphan. Inhibiting NEP with thiorphan resulted in approximately a 1.5-fold increase in MCF-7 cell invasion (Figure 2a) but had no effect on cell viability (Supplementary Figure 3), supporting our hypothesis. Thiorphan had no effect on MDA-MB-231 invasion (data not shown), consistent with our observations that these cells express minimal NEP. Next, we transfected MCF-7 cells with siRNA targeting NEP and control siRNA. RT–PCR confirmed the knockdown of NEP mRNA in the transfected MCF-7 cells (Figure 2b), and these cells invaded significantly more than control MCF-7 cells (Figure 2c). Transfecting an expression vector encoding for NEP in MDA-MB-231 cells efficiently increased mRNA expression levels (Figure 2d) and reduced *in vitro* cell invasion (Figure 2e) compared with cells transfected with the vector alone. Thus, our data suggest that downregulation of NEP in breast cancer cells facilitates invasion.

### NEP regulates invasion through ET receptor signaling

**Figure 3:**
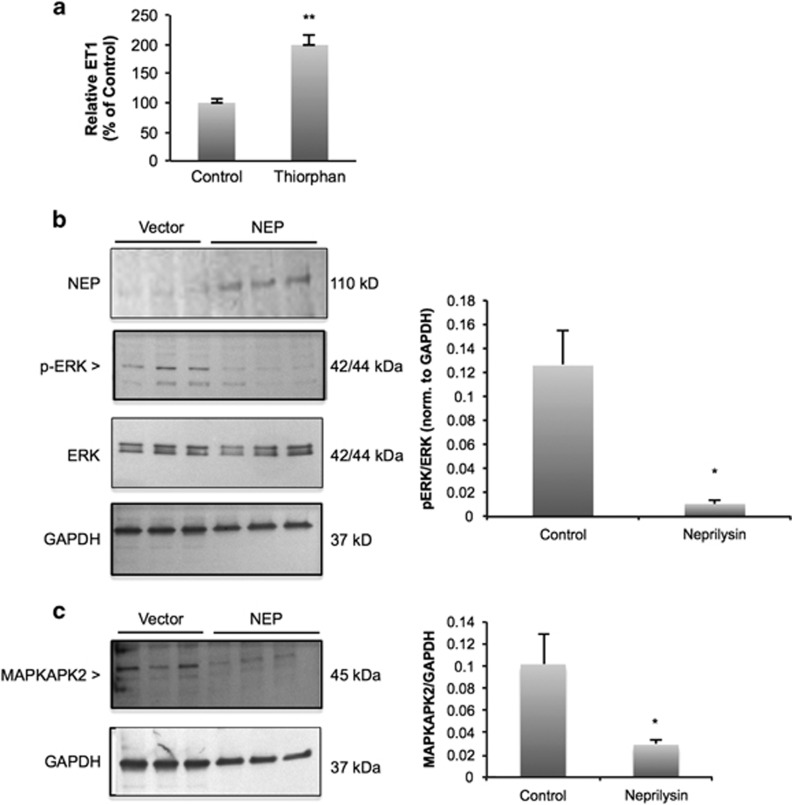
CD10 negatively regulates ERK activation. (**a**) HMEC cells were treated with 25 μg/ml thiorphan (NEP inhibitor), and ET1 levels were measured. Inhibition of NEP increased ET1 levels twofold. Averages are representative of three independent experiments; *P*=0.001. (**b**) Western blotting with MDA-MB-231 cells transfected with NEP show decreased levels of p-ERK compared with controls. Blots are cropped to the appropriate molecular weights for each protein. Semi-quantitative analysis of p-ERK/ERK ratios (both were normalized to GAPDH before calculating the ratio) shows a significant decrease in p-ERK in cells transfected with NEP (*P*=0.029). (**c**) Western blotting with MDA-MB-231 cells transfected with NEP shows decreased levels of total MAPKAPK2 protein relative to GAPDH controls (*P*=0.042).

Because of the predominant role of the ET axis in breast cancer, we next hypothesized that NEP negatively regulates ET-1 levels in breast cancer cells. To address this, we measured ET1 in the supernatants of HMECs treated with the NEP inhibitor thiorphan compared with control HMECs. ET1 peptide levels were relatively low in control-treated HMECs, but treatment with thiorphan resulted in a twofold increase in ET1 levels (Figure 3a).

ET1 binding to the ET A receptor activates the mitogen-activated protein kinase (MAPK) pathway (reviewed by Nelson *et al.*^35^), and phosphorylation of extracellular-signal related kinase (ERK) is a well-known marker MAPK pathway activation. In MDA-MB-231 cells transfected with an expression vector encoding for NEP, western blot analysis revealed a significant decrease in phosphorylated ERK (p-ERK) levels relative to total ERK levels (Figure 3b). In addition, MCF-7 cells transfected with control siRNA demonstrated low levels of phosphorylated ERK, but transfection with NEP-specific siRNA significantly increased p-ERK in these cells (Supplementary Figure 4). Activation of ERK is known to cause increased transcription and translation of mitogen-activated protein kinase-activated protein kinase 2 (MAPKAPK2).^36^ Therefore, we analyzed MAPKAPK2 levels in MDA-MB-231 cells transfected with NEP by western blotting and found that NEP-expressing cells have reduced MAPKAPK2 protein (Figure 3c), consistent with decreased ERK activation. Together, our data suggest that NEP negatively regulates ET1 levels and MAPK signaling in breast cancer cells.

### NEP hypermethylation regulates gene expression and invasion

**Figure 4:**
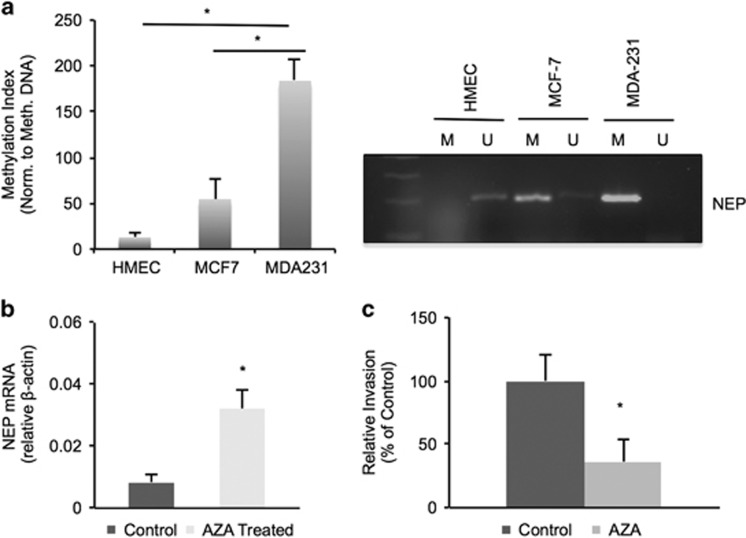
NEP promoter hypermethylation in breast cancer cells regulates expression and invasion. (**a**) Quantitative BSC–PCR from HMEC, MCF-7 and MDA-MB-231 cells shows significantly increased methylation in MDA-MB-231 lines compared with MCF-7 cells (*P*=0.038) or normal HMEC breast cells (*P*=0.033) over three independent experiments. Representative Ct graphs and primer melt curves are shown in Supplementary Figure 5. Right, representative gel from end point BSC–PCR; U=product amplified with unmethylated specific NEP primers; M=product amplified with methylated specific NEP primers. Gels are cropped to the appropriate molecular weight. (**b**) RT–PCR of cDNA from MDA-MB-231 cells treated with AZA shows increased levels of NEP mRNA after AZA treatment (*P*=0.012 over three independent experiments). (**c**) *In vitro* invasion assays with MDA-MB-231 cells treated with AZA or vehicle control show decreased invasion with AZA treatment (*P*=0.0045 over three independent experiments).

We next wished to investigate the mechanism of NEP downregulation in breast cancer cells. Hypermethylation of the NEP promoter has been observed in lymphoid malignancies^37, 38, 39^ and prostate cancer;^40^ we therefore asked whether methylation of the NEP promoter is increased in cancerous cells compared with primary mammary cells. Quantitative bisulfite conversion–PCR (BSC–PCR) analysis of DNA extracted from breast cancer cells with NEP methylation-specific and control beta-actin primers demonstrated that when normalized to control methylated DNA, MDA-MB-231 DNA has significantly higher methylation at the NEP promoter than either MCF-7 or HMEC DNA (Figure 4a; Supplementary Figure 5). A representative gel from end point BSC–PCR depicts the increased methylation in MDA-MB-231 DNA (Figure 4a, right). Treating the highly invasive MDA-MB-231 cells for 5 days with 5-azacytidine (AZA), a DNA methyltransferase inhibitor, resulted in increased NEP mRNA (Figure 4b) and decreased cell invasion compared with vehicle controls (Figure 4c). These results suggest that hypermethylation of the NEP promoter in breast cancer cells facilitates invasion and may represent a novel therapeutic target in breast cancer.

### The NEP promoter is hypermethylated in metastatic human breast tumors

**Figure 5:**
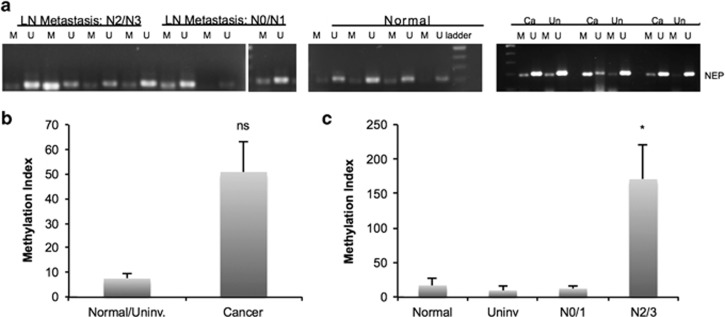
NEP hypermethylation and decreased expression in IDC samples. (**a**) DNA isolated from human IDC tumor samples was subjected to BSC and PCR amplification using methylated specific (‘M') and unmethylated specific (‘U') primers from the NEP promoter. Samples are grouped by lymph node metastasis status; the third N0/N1 sample was run on the same gel as the other samples, but was rearranged for organizational purposes. The normal samples were run on a separate gel but processed and analyzed with the cancer samples. The samples on the right show BSC analysis of matched Cancer (‘Ca') and Uninvolved (‘Un') tissue. Gels are cropped to the appropriate molecular weight. (**b**) Methylation index analysis from quantitative BSC–PCR reveals a non-significant trend of increased methylation in cancer samples compared with normal/uninvolved tissue (*n*=6 for each; *P*=0.09). (**c**) Methylation index from quantitative BSC–PCR analysis reveals that metastatic tumor samples (scored as N2/N3 by lymph node analysis; *n*=3) have significantly higher NEP promoter methylation than normal breast tissue (*n*=3; *P*=0.04), uninvolved breast tissue (*n*=3; *P*=0.039), and cancer tissue with little or no metastasis (scored as N0/N1; *n*=3, *P*=0.039).

To determine whether our observations in cultured breast cancer cell lines are representative of clinical findings, we analyzed primary IDC samples (*n*=6), along with normal and uninvolved breast tissue (*n*=6), from the Cooperative Human Tissue Network (CHTN). We performed BSC–PCR analysis of DNA extracted from these samples and observed increased amplification with the methylation-specific primers in the metastatic breast cancers (N2/3) compared with the poorly metastatic IDC (N0/1) and normal controls (Figure 5a). Quantitative BSC analysis revealed that there was a non-significant trend of increased NEP methylation in cancer samples compared with controls (Figure 5b). Interestingly, we observed a significant increase in methylation in highly metastatic IDC samples when tumors were grouped by lymph node metastasis. Tumors given a lymph node metastasis score (‘N') of 2–3 had an eightfold increase in methylation index compared with normal tissue, uninvolved tissue or cancer tissue with an N score of 0–1 (Figure 5c). Thus, methylation of the NEP promoter is associated with increased IDC metastasis in the samples we tested.

### NEP expression and methylation associate with survival in subtypes of invasive breast cancer

**Figure 6:**
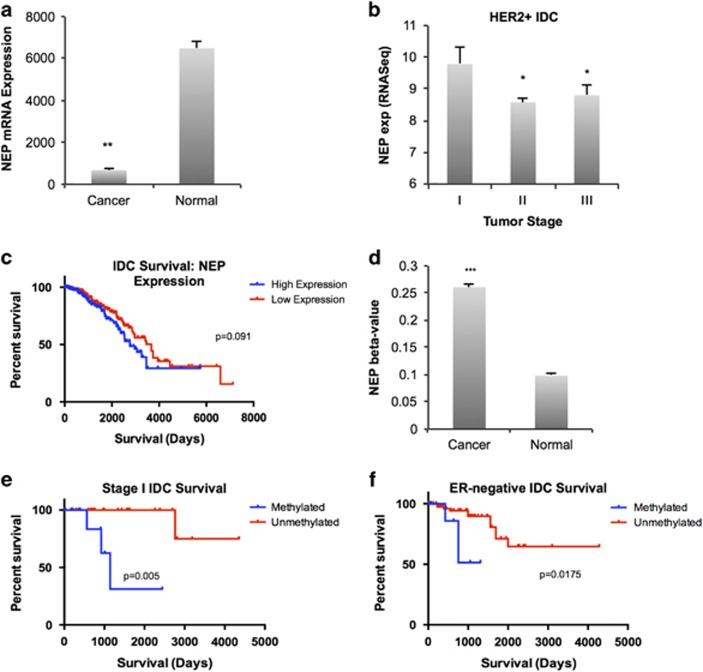
Analysis of NEP expression and methylation in cancer databases. (**a**) NEP expression analysis using human clinical samples of invasive breast cancer available on the MethHC database reveals a statistically significant decrease in NEP in the cancer samples (*P*<0.005, *n*=726 cancer samples, *n*=84 normal samples). (**b**) Invasive breast cancer data from Cancer Browser was sorted for HER2-positive cancers, and NEP expression was analyzed by stage (*n*=67; *P*=0.01 for I vs II and *P*=0.03 for I vs III). (**c**) Kaplan–Meier plot using Kaplan–Meier Plotter of overall survival of invasive breast cancer samples with high (*n*=397) and low (*n*=462) NEP expression (*P*=0.091). (**d**) Analysis of human breast cancer from the MethHC database shows a significant difference between NEP methylation in normal breast tissue (*n*=738) and breast cancer (*n*=90). (**e**) Kaplan–Meier plot generated using Cancer Browser DNA methylation and clinical data shows survival differences between stage I IDC samples with high (*n*=10) and low (*n*=25) NEP promoter methylation (*P*=0.005). (**f**) Kaplan–Meier plot using Cancer Browser DNA methylation and clinical data of IDC samples of ER-negative samples shows significantly improved survival in patients with low NEP promoter methylation (*n*=11) compared with high NEP methylation (*n*=95) (*P*=0.0175).

Next, we analyzed invasive breast cancer using cancer genome databases MethHC^41^ and Cancer Browser.^42^ Comparing expression of the NEP promoter in breast cancer and normal tissue across a large number of invasive breast cancer samples (*n*=726) and normal controls (*n*=84) through the MethHC database revealed a statistically significant decrease in NEP mRNA expression in cancer samples (Figure 6a). To determine whether NEP expression differed within tumor samples, we grouped samples by varying characteristics (ER/PR/HER2 expression and clinical subtype) and analyzed NEP expression levels across cancer stages using RNASeq data from the Cancer Browser database.^42^ We observed that NEP mRNA was significantly higher in stage I HER2-positive IDC than either stage II or stage III cancers (Figure 6b), but no significant differences were seen in other subgroups. We next asked whether NEP expression associated with survival by analyzing survival between high and low NEP-expressing IDC patients using the Cancer Browser database.^42^ No significant difference was observed between these groups (Figure 6c; Table 1). We further sorted cohorts on the basis of ER expression, human epidermal growth factor receptor 2 (HER2), lymph node status, molecular subtype and cancer stage. Unexpectedly, we observed a statistically significant decrease in survival of high NEP-expressing patients with stage I and stage III IDC, but when the cohort was restricted to include only samples with at least 65% tumor cells, thus limiting the stromal contribution, this effect was lost (Table 1).

Next, we analyzed NEP methylation using cancer databases. MethHC analysis revealed significantly higher methylation of the NEP promoter in breast cancer samples (*n*=738) compared with controls (*n*=90) (Figure 6d). Comparing IDC survival rates in cohorts sorted on the basis of NEP promoter methylation with the Cancer Browser database demonstrated that although there was no significant difference in survival across all IDC samples (Table 2), there was a significant survival difference in stage I IDC tumor samples associated with NEP methylation. Stage I IDC samples with NEP promoter methylation had significantly lower survival rates (Figure 6e; Table 2). We did not observe a difference in patients with stage II or stage III IDC (data not shown). In addition, when we sorted cohorts on the basis of ER and HER2 expression, we found that all groups except ER-positive IDC samples showed correlation with methylation status and survival (Table 2). Particularly among ER-negative IDC samples, high NEP promoter methylation correlated with poor survival (Figure 6f; Table 2). Together, these results suggest that NEP methylation could be a useful predictor of survival in stage I and ER-negative IDC.

## Discussion

The present study provides clarity to the contradictory reports of NEP expression in clinical samples of breast tumors. Whereas Smollich *et al.*^11^ reported an association of NEP with disease-free survival and decreased metastasis, many other studies have implicated NEP as a potential biomarker of metastatic cancers.^21, 22, 23, 24, 25^ Our observation that NEP expression is suppressed in invasive breast cancer cells and IDC samples (Figure 1) and negatively regulates breast cancer invasion *in vitro* (Figure 2) are consistent with the clinical studies that correlate NEP expression with a better prognosis.

In a number of the studies concluding that NEP associates with increased metastasis and decreased survival, it is the stromal expression of NEP that predicts a poor prognosis.^21, 22, 24, 33^ In support of this, we observed that in high grade, metastatic IDC samples, NEP expression was decreased and predominately restricted to the stroma, compared with its epithelial expression in low-grade cancers (Figure 1d). In addition, our initial analysis of cancer databases showed that high NEP expression correlated with low survival in both stage I and stage III IDC, but when tumor samples with a high stromal contribution were eliminated from the cohort, this correlation was eliminated (Table 1). This suggests that stromal NEP expression may be the primary contributor to the lower survival rates. Thus, it is feasible that NEP functions differently in tumor cells and stromal cells, and its stromal/epithelial expression could have opposing effects on cancer invasion and metastasis, perhaps by enzymatic cleavage of different substrates. This could help clarify the conflicting reports in the literature on NEP expression correlating with metastasis and survival. Ongoing experiments in our lab aim to further analyze NEP expression and function in tumor and stromal cells.

Our observation that NEP abrogates breast cancer cell invasion (Figure 2) suggests that epithelial-specific reactivation of NEP could serve as a potential future therapy for the treatment of invasive breast cancer, assuming further research supports the utility of this approach. Upregulating NEP has been investigated as an alternative strategy for targeting the ET axis in prostate cancer^7^ and ovarian cancer,^8^ and similar ideas could also be extended to breast cancer therapy. Alternatively, our finding that NEP negatively regulates ERK phosphorylation through the degradation of ET-1 in breast cancer cells (Figure 3) suggests that targeting either the MAPK pathway or the ET axis could mimic reactivation of NEP in breast cancer therapy, particularly in ER-negative breast cancers.

We were prompted to specifically ask whether NEP regulates ET-1 levels and the ET1 receptor-activated MAPK pathway (Figure 3) owing to the predominant role of the ET axis in breast cancer^26, 27, 28, 29, 30, 31^ and found that NEP is a potent regulator of ET1 levels and MAPK signaling (Figure 3). However, the results reported in this study cannot rule out additional NEP substrates that could be regulating breast cancer invasion and progression in either epithelial or stromal cells. For example, substance P, another NEP substrate,^3^ is a known regulator of cell migration, proliferation, invasion and angiogenesis, and neutrophil migration and inflammation (reviewed by Esteban *et al.*^43^ and Shipp *et al.*^44^). Furthermore, NEP cleavage of angiotensin, bradykinin and fibroblast growth factor-2,^4, 45^ all important regulators of angiogenesis, suggests that it could be a regulator of tumor angiogenesis. In support of this, NEP has been reported to inhibit angiogenesis in prostate cancer.^46^ Further studies are needed to determine whether NEP activity toward other substrates regulates epithelial breast cancer cells or the surrounding tumor microenvironment.

Importantly, we found that the NEP promoter is hypermethylated in breast cancer cells and clinical IDC samples compared with normal cells and tissue (Figures 4 and 5). These findings are corroborated by our observation that NEP promoter methylation specifically associated with decreased survival in stage I IDC patients (Figure 6e), suggesting that NEP methylation could be useful in predicting early-stage cancers that are inherently more aggressive and could help inform therapeutic strategies in these patients. NEP promoter methylation also significantly associated with poor survival in ER-negative breast cancers (Figure 6, Table 2). Thus, our findings could have future implications for treating ER-negative/NEP-low breast cancers with MAPK inhibitors or other targets downstream of ET1 signaling. In addition, these results, coupled with the lack of clear correlation between NEP expression and survival, suggest that NEP methylation may be a more effective diagnostic marker than NEP expression in IDC.

In addition, AZA treatment of breast cancer cells reversed NEP suppression while decreasing invasion. A number of clinical trials are being conducted to investigate the therapeutic potential of targeting breast cancers with epigenetic-modifying drugs.^47, 48, 49, 50^ Our studies implicate NEP as an additional genetic target of AZA treatment that would suppress breast cancer invasion. However, because of the potentially negative effects stromal NEP expression has on breast cancer prognosis, it must be carefully determined whether *in vivo* AZA treatment affects stromal NEP expression.

Together, our results support our hypothesis that NEP is an important negative regulator of breast cancer invasion and functions by limiting ET-1 levels and activation of the MAPK pathway. In addition, we provide an epigenetic mechanism of NEP silencing in breast cancer cells, implicating NEP methylation as a possible biomarker and therapeutic target for future breast cancer research.

## Materials and methods

### Cell culture

MCF-7, MDA-MB-231 and HMECs were obtained from ATCC (Manassas, VA, USA). MCF-7 cells were cultured in Eagle's Minimum Essential Medium with 10% fetal bovine serum and 10 μg/ml insulin. MDA-MB-231 cells were cultured in Dulbecco's modified Eagle's medium supplemented with 10% fetal bovine serum. HMECs were cultured with the recommended Mammary Epithelial Cell Growth Medium supplemented with the Mammary Epithelial Cell Growth Kit (ATCC). All cells were used within 1 year of validation and mycoplasma contamination testing from ATCC. All cells were grown at 37 °C and 5% CO_2_.

### Invasion assays

Invasion assays were performed as described previously.^51^ Briefly, diluted Matrigel (1:5; Becton Dickinson, San Jose, CA, USA) was coated on 24-well FluoroBlok invasion inserts (Corning, Inc, Corning, NY, USA). Cells were detached and resuspended to 100 000 cells/ml in serum-free Dulbecco's modified Eagle's medium. One hundred microliters of cell suspension were pipetted into the top chamber, and 600 μl of complete media were pipetted into the lower chamber. Plates were incubated at 37 °C overnight. After removal of cells from the top chamber, inserts were incubated in phosphate-buffered saline/calcein AM (Thermo Fisher Scientific, Waltham, MA, USA) for 30 min and imaged using an inverted fluorescent microscope (Motic Moticam Pro 282B, Richmond, BC, Canada). Fluorescent cells were manually counted and images were recorded. Three independent experiments were performed in triplicate.

### Chemicals, nucleic acids and antibodies

5-AZA was purchased from Sigma–Aldrich (St Louis, MO, USA) and dissolved in a 1:1 solution of acetic acid:water to a concentration of 5 mg/ml, then further diluted with Dulbecco's modified Eagle's medium/10% fetal bovine serum to the working concentration of 5 μM and allowed to buffer at 37 °C before adding to cultured cells at 20% confluence. AZA-containing growth media was replaced daily for 5 days. DL-thiorphan was purchased from Sigma–Aldrich and dissolved in ethanol to 50 mg/ml. A final concentration of 25 μg/ml of the stock solution diluted in water was used for invasion assays. siRNA specific for NEP (sc-29959), along with control siRNA (sc-37007) were purchased from Santa Cruz Biotechnology, Inc. (Dallas, TX, USA). The CD10(NEP)/pCR-TOPO4 cloning vector was obtained from Open Biosystems/Dharmacon (Pittsburg, PA, USA, catalog # MHS1768-101376394). NEP antibody for flow cytometry was purchased from BD Pharmingen (San Jose, CA, USA, catalog # 555375), and NEP antibody for immunohistochemistry was purchased from Novus Bio (Littleton, CO, USA; catalog # NBP-79003). p-ERK (sc-16981), MAPKAPK2 antibody (H-66) and GAPDH (FL-335) antibodies for western blotting were all purchased from Santa Cruz Biotechnology. The total ERK antibody was purchased from Cell Signaling Technology (Danvers, MA, USA; catalog #4659). Secondary antibodies, including goat anti-mouse IgG-HRP and goat anti-rabbit IgG-HRP, were also purchased from Santa Cruz Biotechnology.

### Quantitative reverse transcription–polymerase chain reaction (RT–PCR)

RNA was isolated from cultured cells using Qiagen's RNeasy kit according to the manufacturer's protocol (Qiagen Inc, Valencia, CA, USA). One hundred and twenty nanograms of RNA were then reverse-transcribed using the Bio-Rad iScript cDNA synthesis kit according to the manufacturer's recommended protocol (Bio-Rad Laboratories, Inc, Hercules, CA, USA). Quantitative RT–PCR was conducted with 300 nM ACTB or NEP primers (Supplementary Figure 1) using iQ SYBR Green SuperMix (Bio-Rad) and the CFX Connect Real-Time PCR System (Bio-Rad) according to the recommended protocol. No RT controls were amplified with both primer sets; fold-change over no-RT controls was calculated for each sample. The thermocycler conditions were as follows: 94 °C–3 min, followed by 40 cycles of 94 °C–15 s, 57.5 °C–45 s, 72 °C–45 s, followed by a melt curve analysis. Analysis was performed using the ΔΔC_T_ method.^52^ Cell line analysis was performed over three independent experiments with technical duplicates; IDC analysis was performed with seven tumor samples and four normal controls in technical triplicates. RNA extraction and reverse transcription was performed twice for each sample. Gels from end point RT–PCR were imaged using the Bio-Rad Gel Doc XR+, and semi-quantitative analysis was performed using the Image Lab software (Bio-Rad).

### Western blotting

Halt Protease Inhibitor Cocktail (Life Technologies) was added to Pierce RIPA buffer (Life Technologies), and cells were lysed according to the manufacturer's protocol. Protein lysate was quantified using the Pierce Coomassie Plus Assay kit (Life Technologies) and loaded onto Mini-PROTEAN TGX Gels, 4–20% (Bio-Rad). Gels were transferred to nitrocellulose membranes (Bio-Rad). Western blotting was performed using the Opti-4CN Substrate Kit (Bio-Rad), following the manufacturer's protocol. Membranes were blocked at 4 °C overnight and incubated with primary antibody (1:200 for NEP, p-ERK, MAPKAPK2, 1:1000 for ERK and 1:500 for GAPdH) and secondary antibody (1:2000 for goat anti-rabbit IgG-HRP and 1:3000 for goat anti-mouse IgG-HRP). Detection was performed using the Opti-4CN substrate and the Amplification kit (Bio-Rad), and blots were imaged on the Bio-Rad Gel Doc XR System. Semi-quantitative analysis was performed using the Image Lab software (Bio-Rad).

### NEP cloning

NEP cDNA in a pcr4-TOPO cloning vector (Origene, Rockville, MD, USA) was used as a PCR template to amplify NEP using the NEP cloning primers shown in Supplementary Figure 1. The PCR product was ligated into a pCDNA3.1/NT-GFP-TOPO vector (Life Technologies) and transformed into electro-competent bacteria according to the manufacturer's protocol. Plasmid DNA was purified from transformed bacterial clones using the QiaPrep Spin Miniprep Kit (Qiagen) and sequence-verified.

### Cell transfections

For siRNA transfections, Lipofectamine 2000 (Life Technologies) and 100 pmol sirNA was used according to the manufacturer's recommended protocol. Seventy-two hours following transfection, cells were harvested for RNA extraction or invasion assays. To overexpress NEP, MDA-MB-231 cells were seeded in 6-well plates at approximately 90% confluence, and 7.5 μg of the forward or reverse pCDNA3.1/NEP vector was diluted in Lipofectamine 2000 (Life Technologies) according to the manufacturer's protocol. Forty-eight hours following transfection, cells were harvested for RNA extraction and invasion assays.

### Quantitative BSC–PCR

DNA was isolated from cell lines using Qiagen's DNeasy Blood & Tissue Kit (Qiagen Inc). Two hundred nangrams of genomic DNA were added to each BSC reaction. The EpiTect Bisulfite kit (Qiagen) was used according to the manufacturer's protocol. One microliter of bisulfite-converted DNA was added to each 20 μl PCR reaction along with 10 μl iQ SYBR Green supermix (Bio-Rad) and 300 nM NEP methylation-specific primers or ACTB primers from a CpG-free region (Supplementary Figure 1). The thermocylcer conditions were the same as those described above for quantitative RT–PCR, with annealing temperatures as shown in Supplementary Figure 1. Bisulfite-converted positive control human methylated DNA was used as a calibrator (Thermo Fisher Scientific), and the methylation index was calculated as described previously.^53^ For BSC analysis of cell lines, we conducted three independent experiments with technical duplicates. For BSC analysis of human mammary tissue, six tumor samples, three normal breast controls and three patient-matched uninvolved tissues were tested in technical triplicate. For end point BSC–PCR, Taq Core PCR kit (Qiagen) was used according to the manufacturer's protocol, using the primer sequences originally published by Usmani *et al.*^40^ listed in Supplementary Figure 1.

### Analysis of clinical breast tumors

Tumors representing IDC (*n*=7), matched adjacent normal tissue (*n*=3) and normal breast controls from breast reduction (*n*=4) were provided by the Cooperative Human Tissue Network, a National Cancer Institute supported resource. Other investigators may have received samples from these same tissue specimens. Tumor samples were anonymously coded by the CHTN, and the protocol was approved and exempt status was granted by Lipscomb University's Institutional Review Board. For DNA extraction, ⩽25 mg tissue was processed using Qiagen's DNeasy Blood and Tissue kit (Qiagen Inc). RNA extraction was performed on ⩽150 mg tissue using the Quick RNA MiniPrep Kit according to the manufacturer's protocol (Zymo Research, Irvine, CA, USA). Downstream qBSC–PCR and qRT–PCR were performed as described above.

### Immunohistochemistry

Paraffin slides of tumors (*n*=6) were supplied by the CHTN; immunohistochemistry with a NEP-specific antibody (Novus Bio) was used at 1:200 dilution. Staining was performed by the Vanderbilt Shared Pathology Resource Lab using the manufacturer's recommended protocol. Images were viewed with a × 10 objective and captured with a Canon EOS Rebel T6i camera (Canon, Tokyo, Japan) fixed to a Tritech Research microscope (Tritech Research, Los Angeles, CA, USA). Scale bars represent 100 μm.

### Cancer database analysis

We used the open access MethHC Database (http://methhc.mbc.nctu.edu.tw/php/index.php) to analyze NEP/membrane metallo-endopeptidase expression and methylation in 748 (734 for expression) breast invasive carcinomas and 129 (94 for expression) normal samples.^41^ The Cancer Browser (https://genome-cancer.ucsc.edu)^42^ was used to analyze breast cancer survival and promoter methylation using the Human Methylation27 (*n*=345) and the Illumina HiSeq pancan normalized (*n*=1215) breast invasive carcinoma databases. Data were sorted to analyze only infiltrative ductal carcinoma (IDC), and expression and methylation groups (high and low) were assigned on the basis of values +/− 10% of the mean. Kaplan–Meier survival curves were generated using GraphPad Prism 6, and the Mantel-Cox test was used to determine significance. Hazard ratios were calculated for high methylation/low methylation and high expression/low expression. Confidence intervals (95% CI) of the ratio are reported.

### ET1 ELISA

HMEC cells were detached from culture dishes, resuspended in media and counted. Cells were plated in a 24-well plate at a seeding density of 50 000 cells/well and incubated overnight. Media was then removed and replaced with 500 μl media containing 25 μg/ml thiorphan or incubated with 500 μl media containing 25 μg/ml ethanol as a vehicle control. Cells were incubated for 24 h, then the media was removed and stored at −80 °C. The Endothelin-1 Quantikine ELISA kit (R&D Systems, Minneapolis, MN, USA) was used according to the manufacturer's protocol to quantify ET-1.

### Flow cytometry

Cells were detached from cell culture dishes, rinsed in phosphate-buffered saline, centrifuged and counted. Cells were washed and resuspended in BD Stain Buffer with directly conjugated NEP antibody (1:10) to a concentration of 1 × 10^5^ cells/200 μl and incubated on ice for 45 min. Cells were washed three times and resuspended in 200 μl BD Stain Buffer for flow cytometry analysis. Cells were analyzed on BD Accuri C6 flow cytometer and BD CSampler software (Becton Dickinson and Company, Franklin Lakes, NJ, USA).

### Statistics

All quantitative experiments (including qRT–PCR, qBSC–PCR, western blots and ELISAs) were repeated in at least biological triplicate and technical duplicates, as described under the specific methods. Cell-based assays (including invasion assays, cell transfections and viability assays) were performed in triplicate over a minimum of three independent experiments. Sample size was chosen on the basis of accepted standards prior to performing experiments. Within technical replicates, samples exceeding +/−100% deviation from the mean were excluded from analysis. Quantitative data were normalized to controls, and average relative values over multiple independent experiments were calculated. Numbers of tumor samples are shown in the figures. When noted, two-tailed T-tests were calculated between sets of data assuming unequal variance (type 3) for the strictest statistical criteria; resulting *P*-values are shown in the figure legends. Statistics used in Kaplan–Meier curves are described under ‘Cancer Database Analysis' methods.

## Figures and Tables

**Table 1:** Analysis of NEP expression with survival (Cancer Browser)

*NEP analysis*	*Cohort description*	n	P*-value*	*HR (95% CI)*
Expression	All IDC	NEP high: 397; NEP low: 462	0.091	1.38 (0.97–1.9)
Expression	Stage I IDC	NEP high: 63; NEP low: 52	0.045	4.48 (1.003–20)
Expression	Stage I IDC: ⩽65% tumor cells	NEP high: 159; NEP low: 169	0.1	1.099 (0.628–1.92)
Expression	Stage III IDC	NEP high: 60; NEP low: 74	0.0097	3.38 (1.34–8.5)
Expression	Stage III IDC: ⩽65% tumor cells	NEP high: 34; NEP low: 52	0.663	1.3 (0.4–4.27)

Abbreviations: CI, confidence interval; HR, hazard ratio; IDC, invasive ductal carcinoma; NEP, neprilysin. Table showing analysis of NEP expression with clinical data using the Cancer Browser database.^42^ *P*-values from the Mantel-Cox test and HRs (95% CI of ratio) are shown.

**Table 2:** Analysis of NEP methylation with survival (Cancer Browser)

*NEP analysis*	*Cohort description*	n	P*-value*	*HR (95% CI)*
Methylation	All IDC	M: 87; U: 347	0.194	1.62 (0.77–3.6)
Methylation	Stage I IDC	M: 10; U: 25	0.005	150.4 (8.8–2564)
Methylation	ER-positive	M: 58; U: 232	0.71	0.66 (0.25–1.73)
Methylation	ER-negative	M:11; U: 95	0.0175	24.1 (2.3–250.9)
Methylation	HER2-positive	M: 15; U: 34	0.0155	17.77 (1.73–182.5)
Methylation	HER2-negative	M: 26; U: 110	0.0426	5.25 (1.005–27.3)

Abbreviations: CI, confidence interval; HR, hazard ratio; IDC, invasive ductal carcinoma; M, high NEP methylation; NEP, neprilysin; U, low NEP methylation. Table showing analysis of NEP methylation with clinical data using the Cancer Browser database. *P*-values from the Mantel-Cox test and HRs (95% CI of ratio) are shown.
