# Supplementary figures and images for: Epigenetic suppression of neprilysin regulates breast cancer invasion
Source: Oncogenesis. 2016 Mar 7;5(3):e207–. doi: 10.1038/oncsis.2016.16 (PMC4815048; doi:10.1038/oncsis.2016.16)

A

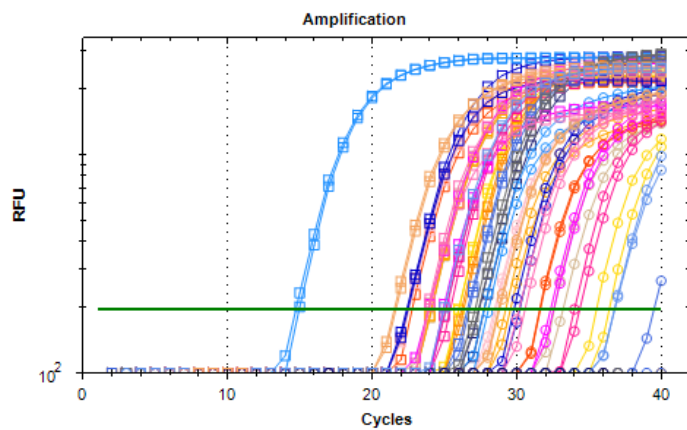

B

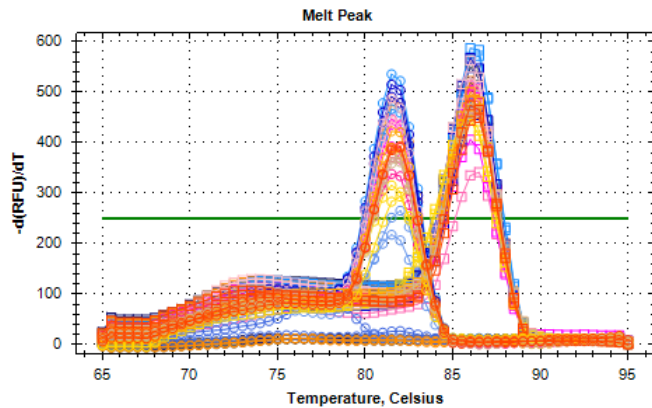

C

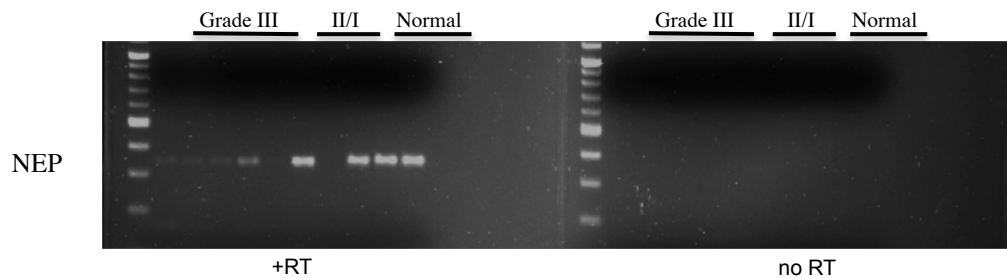

Supplement: Supplementary Figure 1 [file oncsis201616x2.pdf]

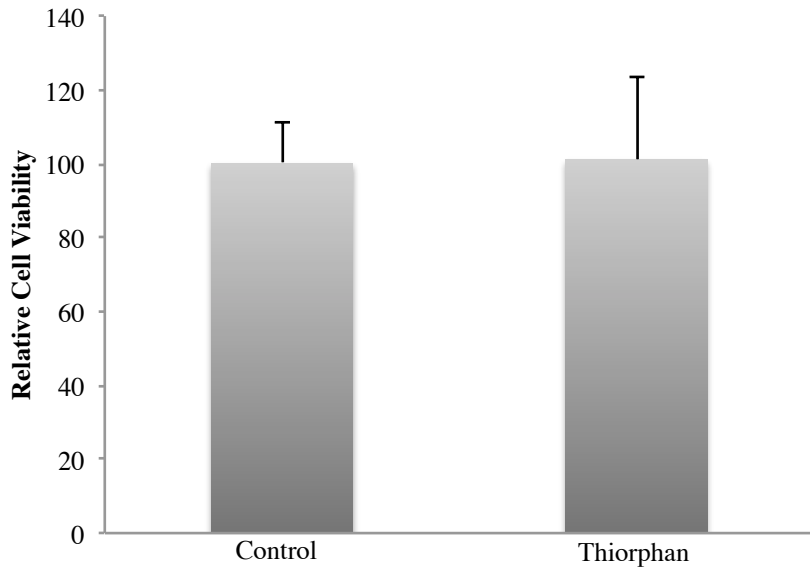

Supplement: Supplementary Figure 2 [file oncsis201616x3.pdf]

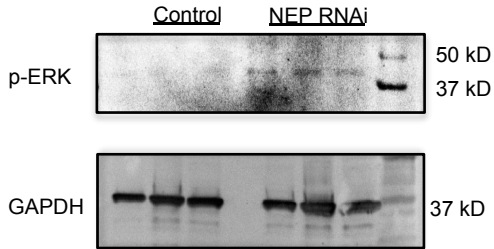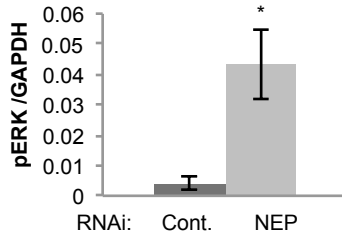

Supplement: Supplementary Figure 3 [file oncsis201616x4.pdf]

A

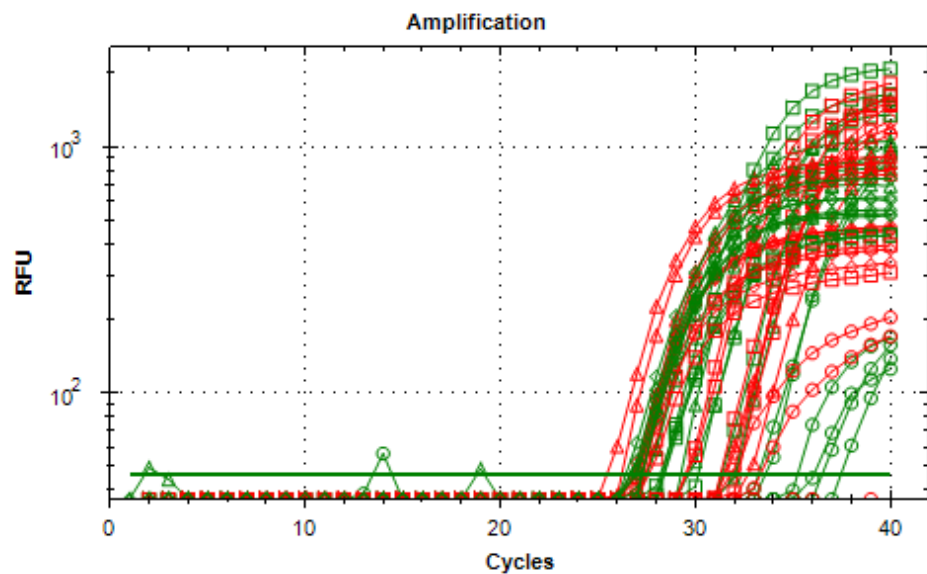

B

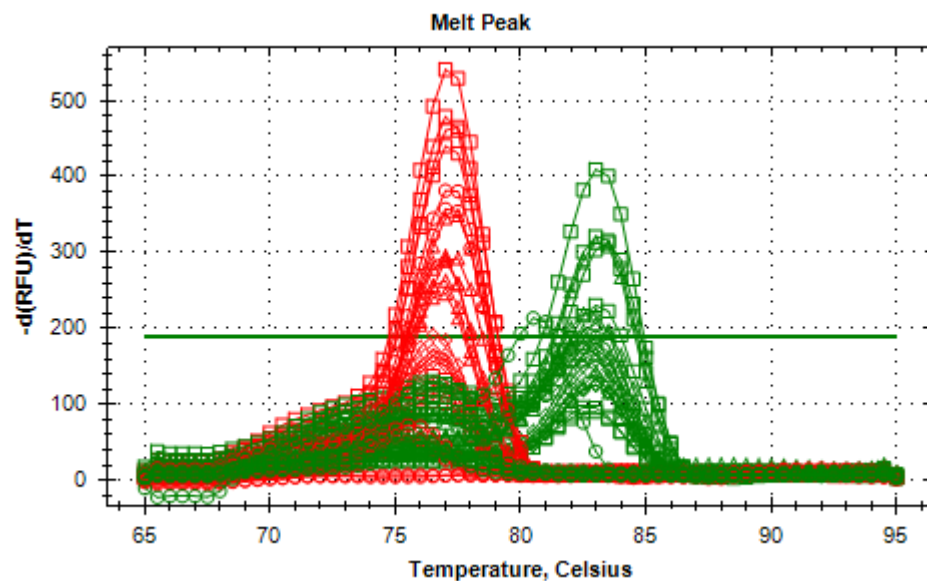

Supplement: Supplementary Figure 4 [file oncsis201616x5.pdf]
