# Supplementary material for: Epigenetic suppression of neprilysin regulates breast cancer invasion
Source: Oncogenesis. 2016 Mar 7;5(3):e207–. doi: 10.1038/oncsis.2016.16 (PMC4815048; doi:10.1038/oncsis.2016.16)
Supplement: Supplementary Figure Legends [file oncsis201616x6.doc]

Supplementary Figure 1 (Excel file). PCR Primer Sequences: All PCR primer sequences and annealing temperatures used in this study are listed in this table.

Supplementary Figure 2 (PDF file). Quantitative RT-PCR Graphs: (A) Representative graph showing log-fluorescent increase by cycle number from the quantitative RT-PCR experiments. Squares represent NEP-amplified products; circles represent ACTB amplified products. Each tumor/normal sample is color-coded (pink shades= tumor; orange/blue shades=normal); (B) Representative melt curve for the ACTB primers (left peak) and the NEP primers (right peak); (C)RT-PCR gel: No-reverse transcription (no-RT) controls run for neprilysin primers with RNA extracted from human tumors (bottom gel; left side is with RT; right side is without RT) demonstrates the specificity of the RT-PCR primers for cDNA.

Supplementary Figure 3 (PDF file). Cell viability graph: MCF-7 cells treated for 24 hours with 25 μg/ml thiorphan or vehicle control and stained with calcein AM to quantitate cell viability show no difference in cell viability with thiorphan treatment.

Supplementary Figure 4 (PDF file). NEP RNAi pERK Western blot: MCF-7 cells transfected with neprilysin-specific RNAi and control RNAi were lysed, and lysates were run on SDS-PAGE gels. Western blotting with p-ERK and GAPDH antibodies shows increased p-ERK levels in the absence of neprilysin.

Supplementary Figure 5 (PDF file). Quantitative BSC-PCR Graphs: (A) Representative graph from the quantitative BSC-PCR experiments. Red indicates ACTB-amplified product; Green indicates NEP-MSP amplified product. Triangle- MDA-MB-231; Circle- HMEC; Square- MCF-7. (B) Representative primer melt curve showing melting temperatures for the products amplified by ACTB primers (red) and NEP-MSP primers (green).
